# Differential responses to doxorubicin-induced phosphorylation and activation of Akt in human breast cancer cells

**DOI:** 10.1186/bcr1259

**Published:** 2005-05-24

**Authors:** Xinqun Li, Yang Lu, Ke Liang, Bolin Liu, Zhen Fan

**Affiliations:** 1 Department of Experimental Therapeutics, The University of Texas MD Anderson Cancer Center, Houston, TX, USA

## Abstract

**Introduction:**

We have shown previously that overexpression of constitutively active Akt or activation of Akt caused by constitutively active Ras or human epidermal growth factor receptor-2 (HER2) confers on breast cancer cells resistance to chemotherapy or radiotherapy. As an expanded study we here report differential responses in terms of phosphorylation and activation of Akt as a result of treatment with doxorubicin in a panel of breast cancer cell lines.

**Methods:**

The levels of Akt phosphorylation and activity were measured by Western blot analysis with an anti-Ser473-phosphorylated Akt antibody and by *in vitro *Akt kinase assay using glycogen synthase kinase-3 as a substrate.

**Results:**

Within 24 hours after exposure to doxorubicin, MCF7, MDA468 and T47D cells showed a drug-dose-dependent increase in the levels of phosphorylated Akt; in contrast, SKBR3 and MDA231 cells showed a decrease in the levels of phosphorylated Akt, and minimal or no changes were detected in MDA361, MDA157 and BT474 cells. The doxorubicin-induced Akt phosphorylation was correlated with increased kinase activity and was dependent on phosphoinositide 3-kinase (PI3-K). An increased baseline level of Akt was also found in MCF7 cells treated with ionizing radiation. The cellular responses to doxorubicin-induced Akt phosphorylation were potentiated after the expression of Akt upstream activators including HER2, HER3 and focal adhesion kinase.

**Conclusion:**

Taken together with our recent published results showing that constitutive Akt mediates resistance to chemotherapy or radiotherapy, our present data suggest that the doxorubicin-induced phosphorylation and activation of Akt might reflect a cellular defensive mechanism of cancer cells to overcome doxorubicin-induced cytotoxic effects, which further supports the current efforts of targeting PI3-K/Akt for enhancing the therapeutic responses of breast cancer cells to chemotherapy and radiotherapy.

## Introduction

Cancer cells with an inherent or acquired capability to resist induction of apoptosis at some point(s) in the signal cascade pathway leading to cell death generally tend to be resistant to chemotherapy or radiotherapy. The serine–threonine protein kinase Akt has received much interest in recent years because it suppresses apoptosis induced by chemotherapy or radiotherapy through interaction with several critical molecules that regulate or execute apoptosis. For instance, after activation, Akt could do the following: it phosphorylates the proapoptotic protein Bcl-2 partner, Bad, which binds to and blocks the activity of Bcl-x, a factor in cell survival [1]; it inactivates caspase-9, which initiates the caspase cascade leading to apoptosis [2]; it represses the forkhead transcription factor FKHRL-1, which regulates the expression of the apoptosis-inducing Fas ligand [3]; and it phosphorylates IκB, thereby promoting the degradation of IκB and increasing the activity of the nuclear factor κB (NFκB) [3,4].

The kinase activity of Akt is triggered after the interaction of its pleckstrin homology domain with the lipid second messenger phosphatidylinositol 3,4,5-trisphosphate, which is generated by phosphoinositide 3-kinase (PI3-K). This interaction recruits Akt from the cytoplasm to the inner cytoplasmic membrane, where Akt undergoes conformational changes and is phosphorylated by the phosphatidylinositol-dependent kinases. The activated Akt is then relocated to the cytoplasm and may be transported further to the nucleus, phosphorylating a wide spectrum of substrates including the molecules mentioned above that are involved in the regulation of cell survival. PI3-K itself is activated by multiple mechanisms, including the activation of growth factor receptor tyrosine kinases [5,6] and G protein-coupled receptors [7,8], integrin-mediated cell adhesion [7,8], and the actions of oncogene products such as Ras [9,10] and hormones such as estrogen [11-13]. By controlling the levels of lipid second messengers, PI3-K regulates various cellular processes, including growth, differentiation, survival, migration and metabolism [14,15].

We have recently shown that expression of a constitutively active Akt, or an increased activity of the human epidermal growth factor receptor-2 (HER2)/PI3-K/Akt or Ras/PI3-K/Akt pathway, leads to multidrug or radiation resistance in human breast cancer cells [16-18]. In those studies we assessed the sensitivity to chemotherapy (including doxorubicin) or radiotherapy of breast cancer cells that contain a higher level of Akt activity due to the overexpression of HER2, constitutively active Ras or constitutively active Akt. To expand our previous studies, we report here a differential pattern of responses of breast cancer cell lines in terms of Akt phosphorylation and activity as a result of treatment with doxorubicin. Depending on the cell types, treatment of breast cancer cells with doxorubicin may trigger a transient phosphorylation and activation of Akt. This therapeutic intervention-triggered activation of Akt depends on an inherent activity of PI3-K, and the capability of the response is potentiated after the expression of Akt upstream regulators including HER2, HER3 or the focal adhesion kinase (FAK), but not by all the signals that are known to affect Akt activity, an example of which is the estrogen-mediated signal. Deprivation of the effect of estrogen did not alter the responsiveness of MCF7 cells to doxorubicin-induced Akt phosphorylation. Our data suggest that the therapeutic intervention-triggered activation of Akt might contribute to the resistance of breast cancer cells to doxorubicin. These results provide further experimental evidence that justifies targeting the PI3-K/Akt pathway to enhance the efficacy of breast cancer chemotherapy or radiotherapy.

## Materials and methods

### Cell lines and cell cultures

Eight breast cancer cell lines used in this study (MCF7, MDA468, SKBR3, MDA157, MDA231, MDA361, BT474 and T47D) were originally purchased from American Type Culture Collection (Manassas, VA, USA). The cells were grown and routinely maintained in Dulbecco's modified Eagle's medium/F12 medium supplemented with 10% fetal bovine serum (FBS), 2 mM glutamine, 100 U/ml penicillin and 100 μg/ml streptomycin. MCF7HER2 cells were described previously [19]. All cells were grown in a 37°C incubator supplied with 5% CO_2 _and 95% air.

### Western blot antibodies and other reagents

Antibodies directed against Akt, Ser473-phosphorylated Akt1 (p-Akt), Ser21/9-phosphorylated glycogen synthase kinase-3 (GSK3), Ser136-phosphorylated Bad and anti-HER2 monoclonal antibody were obtained from Cell Signaling Technology (Beverly, MA, USA). Anti-HER3 antibody was purchased from Santa Cruz Biotechnology (Santa Cruz, CA, USA). Anti-His tag monoclonal antibody was ordered from Upstate Biotechnology (Charlottesville, VA, USA), as was the anti-FAK antibody that recognizes both FAK and FAK-related non-kinase (FRNK), a dominant-negative mutant of FAK [20,21].

The humanized anti-HER2 monoclonal antibody trastuzumab (Herceptin) was made by Genentech (San Francisco, CA, USA). PI3-K-specific inhibitor LY294002 was obtained from CalBiochem (San Diego, CA, USA), and the estrogen receptor (ER) antagonist ICI 182,780 was purchased from Tocris (Ballwin, MO, USA). Doxorubicin (Adriamycin) was ordered from the pharmacy of MD Anderson Cancer Center. All other reagents were purchased from Sigma-Aldrich (St Louis, MO, USA).

### cDNA and transient expression

The pcDNA3 expression construct containing HER3 was provided by Dr Xiaofeng Le (MD Anderson Cancer Center), and the expression constructs of FAK and FRNK (pCMV-Myc) were kindly provided by Dr Thomas Parsons (University of Virginia, Charlottesville, VA, USA). Transient transfection was performed with the FuGENE 6 transfection kit, in accordance with instructions provided by the manufacturer (Roche Diagnostic, Indianapolis, IN, USA).

### Western blot analysis and Akt kinase assay

Western blot analysis and Akt kinase assay were performed as described previously [16,19].

### Cytoplasmic and nuclear fractionation

The method for cytoplasmic and nuclear fractionation was adopted from the literature [22,23] with minor modifications. In brief, pellets containing 2 × 10^7 ^cells were resuspended into 800 μl of buffer A (50 mM NaCl, 10 mM HEPES pH 8.0, 500 mM sucrose, 1 mM EDTA, 0.5 mM spermidine, 0.15 mM spermine, 0.2% Triton X-100, 1 mM phenylmethylsulphonyl fluoride, 2 mM Na_3_VO_4_, 25 μg/ml leupeptin, 25 μg/ml aprotinin). After incubation on ice for 10 min, the cells were homogenized with 10 strokes in a Dounce homogenizer. A small aliquot of the cell homogenates was then examined under a microscope to confirm that more than 98% of cells were lysed. After brief centrifugation of the cell homogenates at 4°C, the supernatant (cytoplasmic fraction) was collected and the pellet was washed twice with 400 μl of buffer B (50 mM NaCl, 10 mM HEPES pH 8, 25% glycerol, 0.1 mM EDTA, 0.5 mM spermidine, 0.15 mM spermine) and then resuspended in 150 μl of buffer C (350 mM NaCl, 10 mM HEPES pH 8.0, 25% glycerol, 0.1 mM EDTA, 0.5 mM spermidine, 0.15 mM spermine) with gentle rocking for 30 min at 4°C [22]. After centrifugation, the supernatant (nuclear fraction) was collected. The amounts of protein in the cytoplasmic and nuclear fractions were determined with the Bradford method (Bio-Rad, Hercules, CA, USA).

### Ionizing radiation

Cells grown on Petri dishes were irradiated with γ-rays from a high-dose-rate ^137^Cs unit (4.5 Gy/min) at room temperature (25 – 27°C), as described previously [17,19]. After irradiation, the cells were harvested by trypsinization.

## Results

### Differential responses in the baseline levels of Akt phosphorylation and kinase activity in a panel of breast cancer cell lines after treatment with doxorubicin

**Figure 1:**
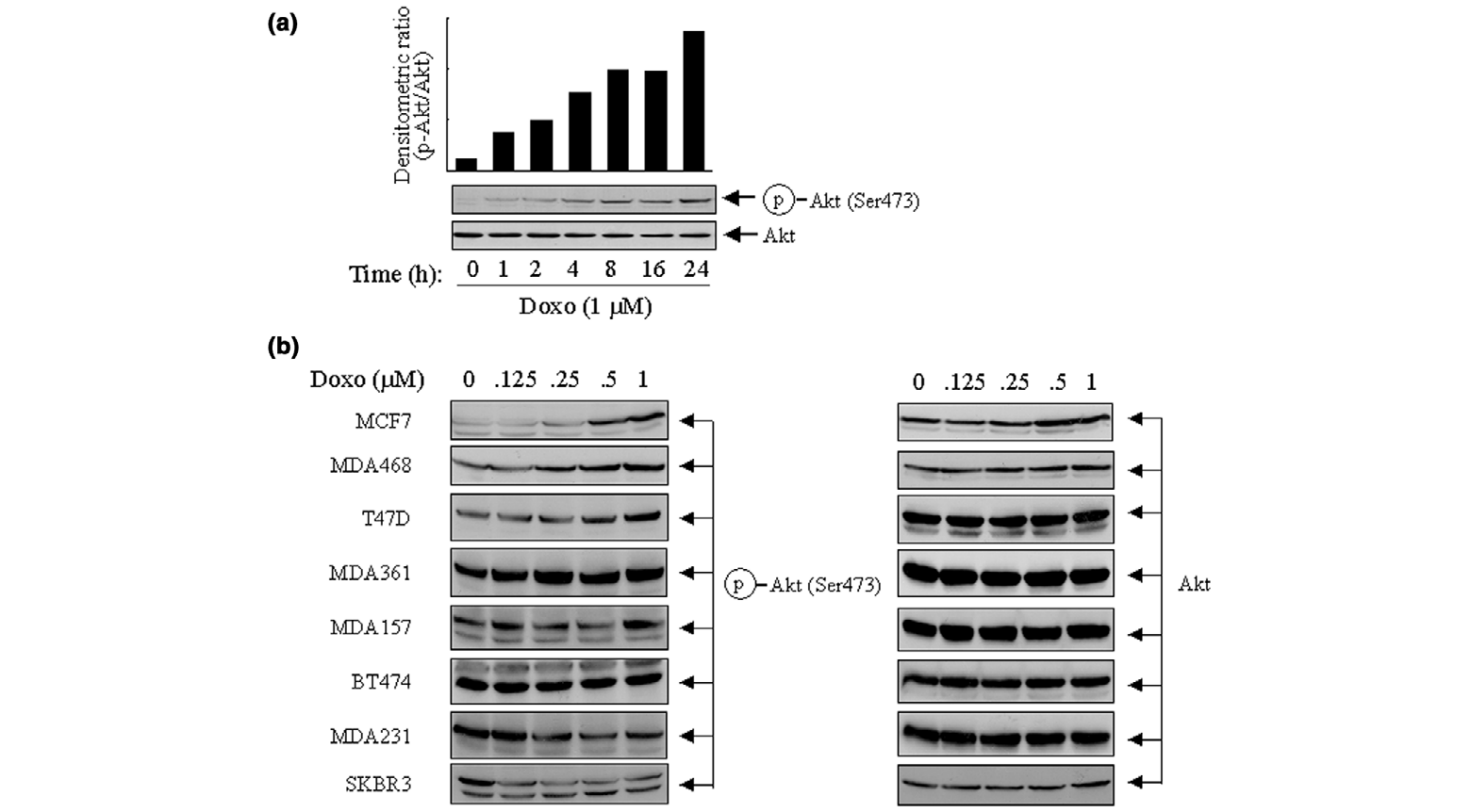
Differential responses to doxorubicin-induced phosphorylation of Akt in a panel of human breast cancer cells. **(a) **MCF7 cells were exposed to 1 μM doxorubicin (Doxo) for the indicated periods in culture medium supplemented with 10% fetal bovine serum (FBS). The cells were then harvested and prepared for Western blot analyses with antibodies directed against Ser473-phosphorylated Akt (p-Akt) and total Akt. The densitometric levels of p-Akt at each time point were normalized to the corresponding levels of total Akt and are shown in the bar graph. **(b) **A panel of eight indicated breast cancer cell lines were treated with 0.125 to 1 μM doxorubicin for 24 hours in culture medium supplemented with 10% FBS. After treatment the individual cell lines were harvested, lysed and subjected to Western blot analyses with antibodies against p-Akt and total Akt.

To assess the cellular responses in breast cancer cells in the baseline levels of Akt phosphorylation and activity as a result of doxorubicin treatment, we first examined the level of Akt phosphorylation and activation in MCF7 breast cancer cells after treatment with doxorubicin. Figure 1a shows a time-dependent induction in the levels of p-Akt with reference to the total levels of Akt in MCF7 cells treated with 1 μM doxorubicin, a dose that we have shown previously to induce apoptosis in the cells [16,18]. An increase in p-Akt level was detected as early as after 1 hour of exposure of the cells to doxorubicin, and a robust increase in the level of p-Akt was observed 24 hours after treatment.

We next expanded the experiment in a panel of eight breast cancer cell lines treated with increasing concentrations (0.125 to 1 μM) of doxorubicin for 24 hours in culture medium supplemented with 10% FBS. Interestingly, it was found that the changes in the levels of p-Akt varied between the cell lines after the treatment. In comparison with control cells, which were kept untreated for 24 hours in the same type of culture medium, MCF7, MDA468 and T47D cells showed a dose-dependent increase in p-Akt levels; in contrast, SKBR3 and MDA231 cells showed a dose-dependent decrease, and no or minimal change was detected in MDA361, MDA157 and BT474 cells (Fig. 1b). As expected, no changes in total Akt expression were found in the cell lines after the treatment. These results suggest that genetic context among individual cell lines might have a role in determining the cellular responses to the treatment.

**Figure 2:**
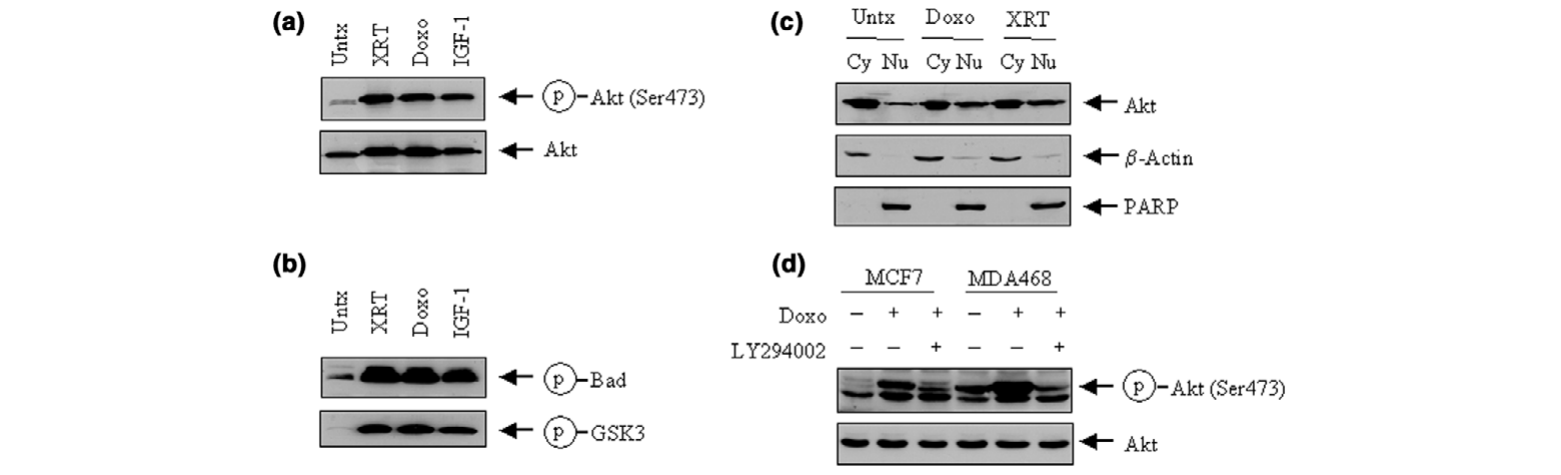
Functional analysis of Akt phosphorylation in human breast cancer cells after treatment with doxorubicin. **(a) **MCF7 cells were exposed to 1 μM doxorubicin (Doxo) for 24 hours, or irradiated (XRT) with X-rays (5 Gy) and then cultured for a further 24 hours in culture medium supplemented with 10% fetal bovine serum (FBS). MCF7 cells stimulated for 15 min with 10 nM type 1 insulin-like growth factor (IGF-1) were used as a positive control. Untreated (Untx) and the treated MCF7 cells were harvested, lysed and subjected to Western blot analyses with antibodies against Ser473-phosphorylated Akt (p-Akt) and total Akt. **(b) **MCF7 cells transiently transfected with a His-tagged Akt1 expression construct were left untreated or treated as in (a). Akt1 was immunoprecipitated by using an anti-His tag monoclonal antibody and then assayed for *in vitro *kinase activities for phosphorylation on Bad and glycogen synthase kinase-3 (GSK3). **(c) **Cytoplasmic and nuclear fractionations from untreated, doxorubicin-treated and γ-ray-irradiated MCF7 cells were separated as described in Materials and Methods. Equal amounts (40 μg) of cytoplasmic fraction (Cy) and nuclear fraction (Nu) from each sample were subjected to Western blot analysis to determine the distribution of Akt after treatment with doxorubicin or ionizing radiation. β-Actin and poly(ADP-ribose) polymerase (PARP) were used as markers of the cytoplasmic and nuclear fractions, respectively. **(d) **MCF7 or MDA468 cells were pre-exposed to 10 μM LY294002 for 2 hours before treatment with 1 μM doxorubicin for 24 hours. After treatment the cells were harvested, lysed and subjected to Western blot analyses with antibodies against p-Akt and total Akt.

To confirm that the phosphorylation of Akt induced by doxorubicin was associated with an increased Akt kinase activity, we assessed Akt activity by *in vitro *Akt kinase assay on two known Akt substrates, Bad and GSK3, in MCF7 cells. Figure 2a shows that, in comparison with untreated MCF7 cells and with the cells treated with type 1 insulin-like growth factor (IGF-1), the cells treated with doxorubicin contained an increased level of p-Akt, which was comparable to the increase of p-Akt level stimulated by IGF-1. Treatment of the cells with ionizing radiation induced a similar increase in the level of p-Akt. The increases in p-Akt level induced by doxorubicin or radiation were associated with increased Akt kinase activities measured by the Akt *in vitro *kinase assay (Fig. 2b). We found that the Akt protein immunoprecipitated from doxorubicin-treated or γ-ray-irradiated cells phosphorylated both Bad and GSK3 as strongly as the Akt protein from the IGF-1-treated cells.

As another measure of the functional status of Akt after treatment with doxorubicin or ionizing radiation, we also examined the translocation of Akt from the cytoplasm to the nucleus. To allow the detection of the signals of Akt from cytoplasmic to nuclear translocation, we raised the level of Akt expression in MCF7 cells by transient transfection of the cells with a His-tagged Akt1 expression construct 48 hours before harvest. Both the doxorubicin-induced and radiation-induced increases in Akt phosphorylation were associated with increased translocation of Akt from the cytoplasm to the nucleus (Fig. 2c).

To determine the extent to which the doxorubicin-induced activation of Akt is regulated by the PI3-K pathway, we explored this question with MCF7 cells, which express a relatively low baseline level of p-Akt, and MDA468 cells, which express a relatively high baseline level of p-Akt because of the mutation status of PTEN (phosphatase and tensin homolog deleted on chromosome ten) in the cells [24]. We found that an overnight (16 hours) exposure of the cells to LY294002, a PI3-K-specific inhibitor, remarkably abolished the increase in Akt phosphorylation after treatment with doxorubicin in both MCF7 and MDA468 cells (Fig. 2d). The results indicate that the doxorubicin-induced phosphorylation and activation of Akt were mediated through a PI3-K dependent pathway.

### Roles of HER family members in doxorubicin-induced activation of Akt

**Figure 3:**
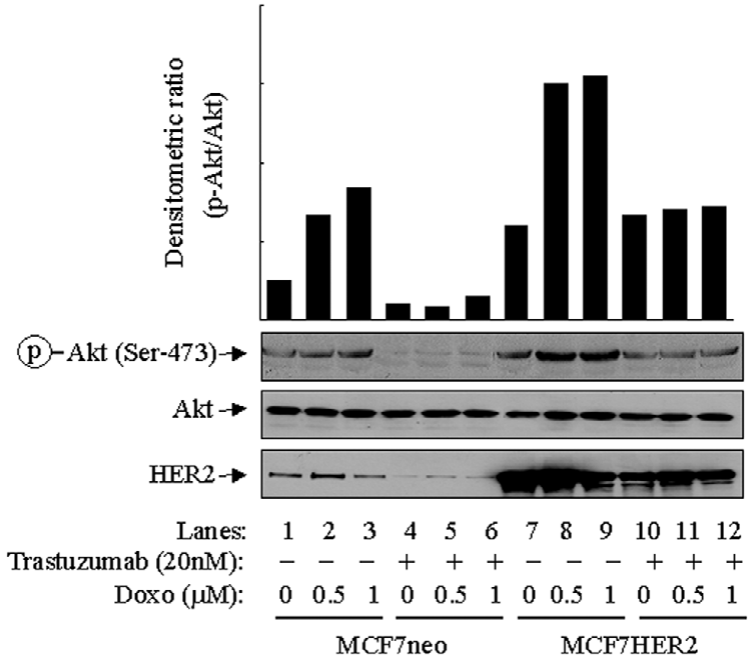
Potentiation to doxorubicin-induced Akt phosphorylation in MCF7 cells after expressing high levels of HER2. Control vector-transfected MCF7 cells (MCF7neo) and MCF7 transfectants expressing high levels of human epidermal growth factor receptor-2 (MCF7HER2) were left untreated or were treated with 0.5 or 1 μM doxorubicin (Doxo) for 24 hours. Separate dishes of MCF7neo and MCF7HER2 cells were pretreated with 20 nM trastuzumab (Herceptin) 24 hours before treatment with doxorubicin. After treatment the cells were harvested, lysed and subjected to Western blot analyses with antibodies against HER2, Ser473-phosphorylated Akt (p-Akt) and total Akt. The densitometric levels of p-Akt were normalized to the respective levels of total Akt in each lane and are shown in the bar graph.

Because the doxorubicin-induced activation of Akt is dependent on PI3-K activity, we proposed that the breast cancer cells with compelling molecular components of the PI3-K pathway might show an enhanced cellular response to doxorubicin-induced activation of Akt. The HER family members are important upstream regulators of the PI3-K/Akt pathway and are known to be important in the progression of breast cancer and its resistance to chemotherapy or radiotherapy [25,26]. To determine the extent to which HER family members might potentiate the cellular response to doxorubicin-induced activation of Akt in breast cancer cells, we assessed the effect of treatment with doxorubicin (0.5 to 1 μM) on p-Akt levels in MCF7 cells transfected with a HER2 expression construct (MCF7HER2 cells). In comparison with control vector-transfected MCF7 cells (MCF7neo), MCF7HER2 cells showed not only a higher baseline level of p-Akt (Fig. 3, compare lanes 1 and 7) but also an enhanced response to the doxorubicin-induced increase in Akt phosphorylation (Fig. 3, compare lanes 1 to 3 with lanes 7 to 9). A caveat is that it is unlikely that the enhancement was caused by an additive effect of Akt phosphorylation by doxorubicin treatment and HER2 overexpression in the cells, because treatment of MCF7neo cells with trastuzumab also decreased the level of doxorubicin-induced phosphorylation of Akt. As expected, we detected no changes in the level of total Akt. The increase in the levels of p-Akt in MCF7neo and MCF7HER2 cells by doxorubicin was markedly diminished by pretreatment with trastuzumab, which downregulates HER2 in these cells (Fig. 3, compare lanes 1 to 3 with lanes 4 to 6, and lanes 7 to 9 with lanes 10 to 12). Taken together, these results indicate that the higher level of HER2 in MCF7HER2 cells potentiates the response of the cells to doxorubicin-induced activation of Akt.

**Figure 4:**
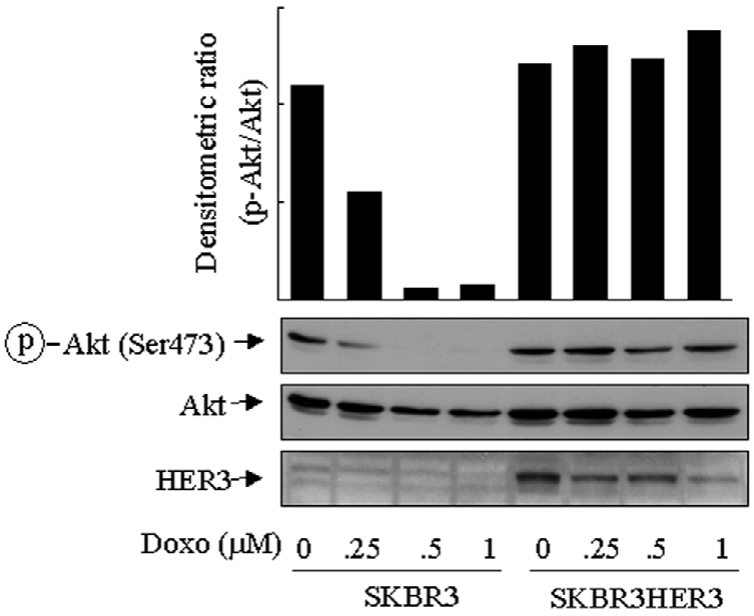
Effect of HER3 expression on decrease in Akt phosphorylation in SKBR3 cells after doxorubicin treatment. SKBR3 cells were transiently transfected with a control vector (pcDNA3) or a HER3 expression construct for 24 hours, and then exposed to 0.125 to 1 μM doxorubicin for a further 24 hours. After treatment the cells were harvested, lysed and subjected to Western blot analyses with antibodies against HER3, Ser473-phosphorylated Akt (p-Akt) and total Akt. The densitometric levels of p-Akt were normalized to the respective levels of total Akt in each lane and are shown in the bar graph.

Interestingly, some cell lines including SKBR3 cells showed a decline in the level of p-Akt after treatment with doxorubicin (Fig. 1), despite the fact that SKBR3 cells express an appreciable level of HER2 [27,28]. A notable difference between MCF7 and SKBR3 cells is that the former expresses HER3 whereas the latter has no detectable level of HER3 expression [16]. Of the HER family members, HER3 contains the most PI3-K-binding sites, but it is kinase-deficient and is mainly activated though heterodimerization with other HER members [29]. We proposed that an insufficient level of HER3 expression might affect the response of SKBR3 cells to treatment with doxorubicin. To test this hypothesis we transiently transfected SKBR3 cells with a HER3 expression construct. Figure 4 shows that, in comparison with control vector-transfected SKBR3 cells, transient expression of HER3 prevented the decline in the level of p-Akt after doxorubicin treatment in SKBR3 cells. It is noteworthy that, in this particular experiment, HER3 was only transiently transfected into the SKBR3 cells, with an estimated 10 to 15% transfection efficiency. Given the result from the mixed (transfected and untransfected) cells, it is reasonable to speculate that selected clonal or pooled HER3-expressing SKBR3 cells would exhibit a pattern of response similar to that observed in MCF7 cells.

Exposure of the transiently transfected cells to doxorubicin also led to a decrease in the level of HER3, the mechanism of which is unknown. We speculate that it might be related to a degradation of the protein after heterodimerization with HER2. Nevertheless, the transient expression of HER3 in only a small fraction of the cell population (10 to 15%; data not shown) prevented the decline in p-Akt after treatment with doxorubicin in a HER2-overexpressing cell line (SKBR3) suggests a potential cooperative role of HER2 and HER3 in the increase in Akt activity after treatment with doxorubicin. Thus, the ability of HER2 to potentiate the cellular response of Akt phosphorylation or activation after treatment with doxorubicin depends on the cell types.

### Involvement of FAK in doxorubicin-triggered phosphorylation and activation of Akt

**Figure 5:**
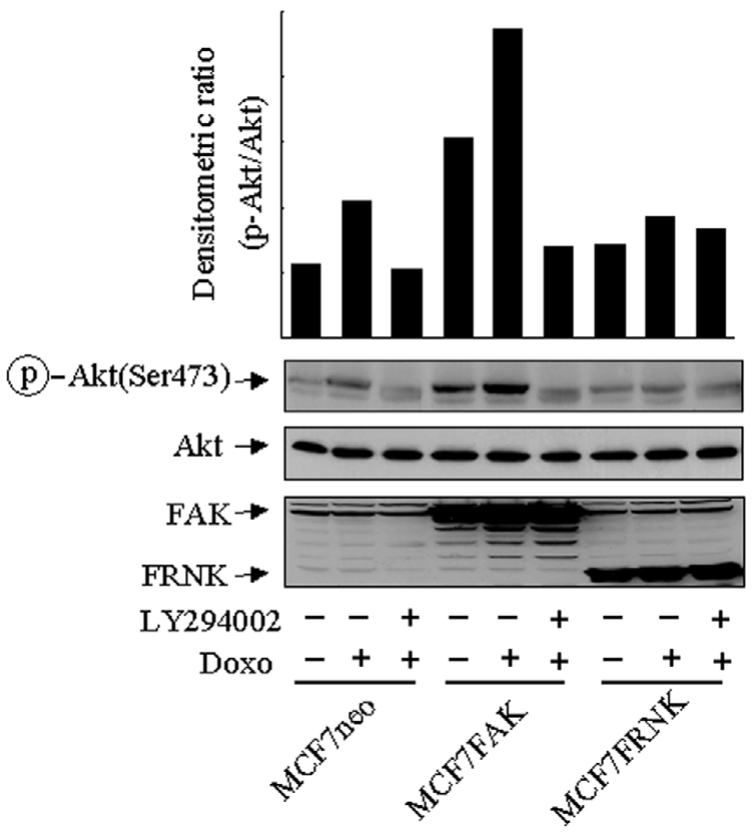
Potentiation of cellular response to doxorubicin-induced Akt phosphorylation in MCF7 cells by FAK. MCF7 cells were transiently transfected for 24 hours with a control vector (pcDNA3.1), a focal adhesion kinase (FAK) expression construct or a FAK-related non-kinase (FRNK) expression construct, followed by exposure to 1 μM doxorubicin (Doxo) for a further 24-hour of culture, with or without the addition of the phosphoinositide 3-kinase-specific inhibitor LY294002 (20 μM) 16 hours before the end of exposure to doxorubicin. After treatment the cells were harvested, lysed and subjected to Western blot analyses with antibodies against FAK, FRNK, Ser473-phosphorylated Akt (p-Akt) and total Akt. The densitometric levels of p-Akt were normalized to the respective levels of total Akt in each lane and are shown in the bar graph.

To broaden the implication of our findings, we sought to assess possible roles of other signal pathways that might also potentiate the cellular response of Akt phosphorylation of MCF7 cells after treatment with doxorubicin. In addition to the HER family members, the FAK pathway is also known to modulate the PI3-K pathway. The FAK pathway is regulated by the interaction between extracellular matrix receptors and integrins, and is often augmented in human breast cancer cells [30,31]. We therefore transiently transfected MCF7 cells with an expression construct of FAK or its dominant-negative counterpart, FRNK. In comparison with control vector-transfected cells, which exhibited a LY294002-sensitive increase in the level of p-Akt over baseline, FAK-transfected cells had a higher p-Akt level both at baseline and after treatment with doxorubicin and were sensitive to LY294002 (Fig. 5). In contrast, transfection of MCF7 cells with FRNK led to a lower phosphorylation level of Akt after treatment with doxorubicin. Irrespective of the expression of FAK or FRNK, the level of total Akt remained unchanged. Taken together, these results suggest that molecular components, such as FAK and HER3, that enhance the cellular sensitivity and responsiveness for PI3-K activation might potentiate the cellular responses of Akt phosphorylation to treatment with doxorubicin.

### Effects of estrogen on doxorubicin-induced phosphorylation and activation of Akt

**Figure 6:**
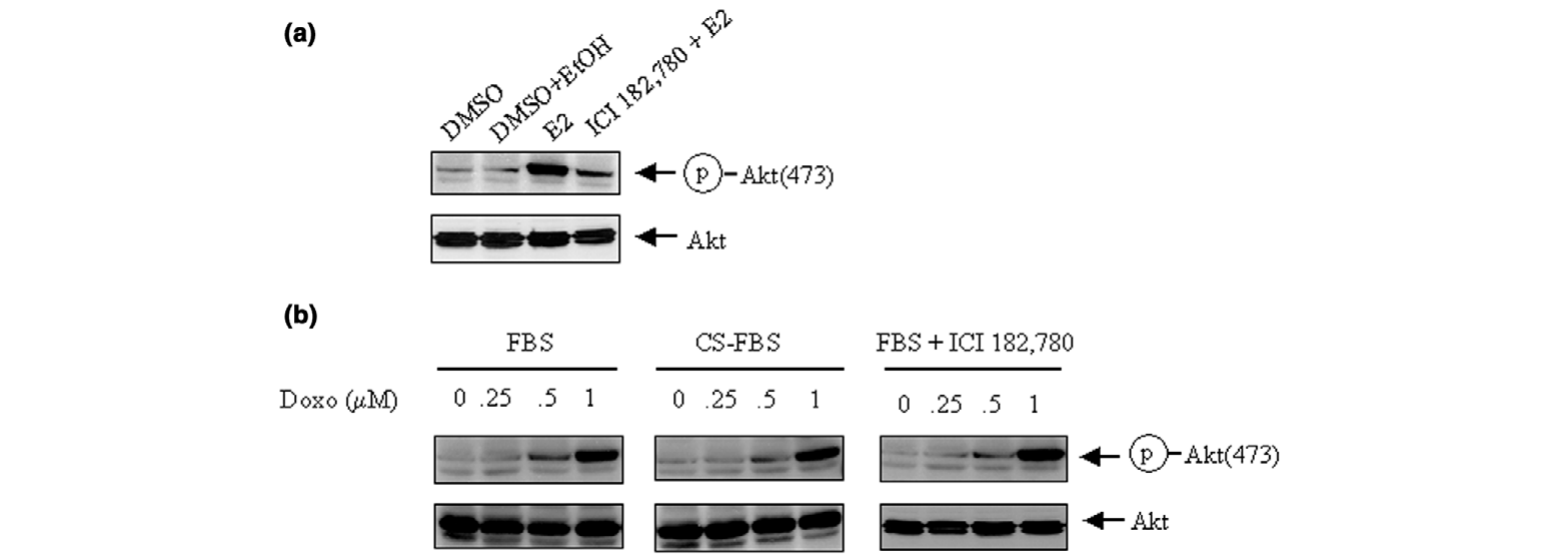
Potential involvement of estrogen-mediated signaling on doxorubicin-induced Akt phosphorylation. **(a) **MCF7 cells grown to subconfluence were exposed to 1 μM ICI 182,780 (an estrogen receptor antagonist) or the vehicles (dimethyl sulfoxide [DMSO] and ethanol [EtOH]) for 30 min, and then treated with or without 10 nM estradiol (E2) for a further 30 min. Cell lysates were prepared for Western blot analysis with antibodies against Ser473-phosphorylated Akt (p-Akt) and total Akt. **(b) **MCF7 cells were cultured in regular 0.5% fetal bovine serum (FBS) medium, charcoal-stripped (CS) FBS medium, or regular 0.5% FBS plus 1 μM ICI 182,780. The cells were then treated with 0.25 to 1 μM doxorubicin (Doxo) for 24 hours. After treatment the cells were harvested, lysed and subjected to Western blot analyses with antibodies against p-Akt and total Akt.

To determine whether the signaling pathways known to modulate the activity of PI3-K/Akt might unanimously potentiate the cellular response of Akt phosphorylation to treatment with doxorubicin, we examined the effect of doxorubicin on the level of p-Akt in MCF7 cells cultured in medium supplemented with an ER antagonist or in estrogen-depleted medium. Estrogen is known to be involved in the regulation of Akt phosphorylation in both ER-positive and ER-negative breast cancer cells [12,32,33]. In comparison with vehicle-treated cells, MCF7 cells stimulated with estrogen (estradiol) showed a higher level of p-Akt, which was decreased when an ER antagonist (ICI 182,780) was present in the culture medium (Fig. 6a). In contrast with the results shown in Figs 4 and 5, we observed no difference in the levels of p-Akt after doxorubicin treatment in MCF7 cells cultured in regular 0.5% FBS medium, charcoal-stripped FBS (that is, estrogen-depleted) medium, or regular 0.5% FBS medium plus ICI 182,780 (Fig. 6b). These results suggested that at least the PI3-K signaling regulated by estrogen does not potentiate the cellular responsiveness to doxorubicin-induced phosphorylation of Akt.

## Discussion

In our present study we found that the activity of Akt, an important signal molecule that promotes cell survival and confers cellular resistance to chemotherapy and radiotherapy as shown by us [16,18,19] and others [34,35], was transiently elevated in a subset of breast cancer cell lines as a result of exposure to doxorubicin, a chemotherapeutic agent commonly used to treat patients with breast cancers. Activation of Akt in MCF7 cells after exposure to doxorubicin was reported earlier, but the mechanism was not explored in detail [34,35]. We noted here that, in comparison with resting (non-stimulated) cells, in which most Akt was found in the cytoplasm, exposure of the cells to doxorubicin or ionizing radiation led to a relocation of Akt to the nucleus. It is noteworthy that several antiapoptotic substrates of Akt are nuclear proteins. This subcellular translocation of Akt is important for cells to overcome the death signals initiated by treatment with doxorubicin or ionizing radiation. Taken together with our previous results [16-18], the present results suggest that doxorubicin-triggered activation of Akt has a role in the resistance of breast cancer cells to this drug and that the same might apply to radiotherapy.

Because the overall cellular sensitivity of breast cancer cells to chemotherapy or radiotherapy is attributed to multiple intrinsic and extrinsic factors, such as p53 status, Bcl-2/Bax levels, expression of multiple drug resistance proteins, and hypoxic status, a caveat is that our data do not necessarily imply that one group of breast cancer cells showing increases in the level of p-Akt after chemotherapy or radiotherapy would absolutely be more chemoresistant or radioresistant than the another group of breast cancer cells without showing such a response. Rather, the data indicate that the activation and phosphorylation of Akt triggered by chemotherapy or radiotherapy contribute to the overall cellular sensitivity to these conventional therapies.

Several questions remain to be fully answered. First, why was Akt activation after treatment with doxorubicin found in only some of the breast cancer cell lines we tested? Apparently, cells must be equipped with certain molecular components that enable them to react to signals induced by chemotherapy or radiotherapy. We found that the drug-triggered activation of Akt depends on the activity of PI3-K, which can be activated by several known pathways, some of which we have explored in the present study. Which pathway is activated depends on the genetic context and functional status of the signal transduction network in individual cell types. In our study, MCF7 cells transiently expressing a high level of HER2 potentiated the response of the cells to the doxorubicin-induced activation of Akt. This result is consistent with those shown recently by us [16,19] and others [36-38] indicating that HER2 expression in breast cancer cells might render them more resistant to chemotherapy or radiotherapy.

However, a high level of HER2 expression alone might not be sufficient to mediate this response. For example, we detected no change in the level of p-Akt in BT474 breast cancer cells after treatment with doxorubicin, even though they expressed a high level of HER2. SKBR3, another breast cancer cell line that expresses high levels of HER2, even showed a reduced level of p-Akt after treatment with doxorubicin. Expression of a transient transfected HER3 in the SKBR3 cells prevented this decline, indicating that heterodimerization and crosstalk between HER2 and HER3 might be important in mediating the downstream pathway that leads to Akt activation in breast cancer cells after treatment with doxorubicin. This might explain the negative findings from a recent clinical study reporting that HER2 overexpression does not seem to predispose to locoregional recurrence for breast cancer patients treated with neoadjuvant doxorubicin-based chemotherapy, mastectomy and radiotherapy [39].

A second question is what molecular executioner leads to the activation of Akt after chemotherapy or radiotherapy. Are any soluble factors or non-secreted membrane-bound ligands involved, or is the PI3-K/Akt pathway activated directly and autonomously? In our study, we demonstrated that several different mechanisms, two of which are the expression of HER2 and of FAK, might enhance the doxorubicin-induced activation of Akt. Each mechanism activates PI3-K but does so through different ligands. Interference with these pathways by the anti-HER2 monoclonal antibody trastuzumab or by a dominant-negative mutant FAK (FRNK) abolished the drug-triggered activation of Akt mediated by HER2 and FAK, respectively. An interesting finding from our studies is that not all stimuli that lead to PI3-K activation enhance the drug-triggered activation of Akt. For example, abnormal estrogen exposure is associated with an increased risk of breast cancer, and estrogen is known to activate Akt via a non-nuclear estrogen-signaling pathway involving the direct interaction of ER with PI3-K [40].

The ER isoform ERα binds to the p85α regulatory subunit of PI3-K in a ligand-dependent manner. Stimulation with estrogen increases ERα-associated PI3-K activity, leading to the activation of Akt. This interaction between ER and p85α is independent of gene transcription and does not involve phosphotyrosine adapter molecules or Src-homology domains of p85α [40]. We found that the ER antagonist ICI 182,780 blocked estrogen-induced Akt activation in the ER-positive MCF7 cells but did not affect doxorubicin-induced Akt activation. Depletion of estrogen from the culture medium did not affect the doxorubicin-induced activation of Akt either. These data suggest that estrogen-induced signals, whether dependent on ER or not, are not involved in the pathway that enhances the doxorubicin-induced activation of Akt.

In fact, this atypical activation of Akt seems not to be limited to doxorubicin or ionizing radiation. We have observed that treatment of MCF7 cells with several different drugs (paclitaxel, 5-flurouracil and gemcitabine) that act through different mechanisms can also induce Akt phosphorylation, although the response and the timing and dose required for this effect varied between the drugs tested (data not shown). Cellular stress such as hypoxia and ultraviolet radiation has been reported by others to induce PI3-K-dependent Akt activation [41-43]. Thus, inherent properties of individual cell types, rather than specific cell death signals, might determine whether Akt is activated after cells are exposed to stresses. Cancer cells with functional aberrations, such as overexpression of HER family members or increased cell adhesion potential, are probably more capable than noncancerous cells of activating Akt as a defensive mechanism against external detrimental stimuli, which justifies a novel approach of targeting the PI3-K/Akt for chemosensitization or radiosensitization.

In summary, doxorubicin might cause a PI3-K-dependent increase of Akt activity in breast cancer cells. Together with other recent results of ours [16-18], the present observations suggest that clinical benefits in treating patients with breast cancer could be obtained with appropriate combinations of novel Akt inhibitors and conventional chemotherapeutic drugs or ionizing radiation.

## Conclusion

We found that the activities of Akt are increased in selected cell lines treated with doxorubicin, which is a PI3-K-dependent process and is potentiated after overexpression of HER2/HER3 receptor tyrosine kinases or FAK nonreceptor tyrosine kinase. This therapeutic intervention (doxorubicin)-triggered activation of Akt might have a role in affecting the overall therapeutic responses of cancer cells to the treatment. Clinical benefits in the treatment of breast cancer patients could be obtained with appropriate combinations of novel Akt inhibitors and conventional chemotherapeutic drugs or ionizing radiation. Our observations further justify the efforts of targeting PI3-K/Akt for enhancing the therapeutic responses of breast cancer cells to the conventional therapies.

## Abbreviations

ER = estrogen receptor; FBS = fetal bovine serum; FAK = focal adhesion kinase; FRNK = FAK-related non-kinase; GSK3 = glycogen synthase kinase-3; HER2 = human epidermal growth factor receptor-2; IGF-I = type 1 insulin-like growth factor; p-Akt = Ser473-phosphorylated Akt; PI3-K = phosphoinositide 3-kinase.

## Competing interests

The author(s) declare that they have no competing interests.

## Authors' contributions

The authors' contributions to this research are reflected in the order shown, with the exception of ZF, who supervised all aspects of this research and prepared the manuscript. XL did most Western blot analyses and sample preparations; YL contributed the experiments of cell transfection and some Western blot analyses; KL performed radiation of the cells; BL participated in the overall design of experiments and data interpretation. All authors read and approved the final manuscript.
